# LncRNA TP73-AS1 sponges miR-141-3p to promote the migration and invasion of pancreatic cancer cells through the up-regulation of BDH2

**DOI:** 10.1042/BSR20181937

**Published:** 2019-03-15

**Authors:** Xian-Ping Cui, Chuan-Xi Wang, Zhi-Yi Wang, Jian Li, Ya-Wen Tan, Song-Tao Gu, Cheng-Kun Qin

**Affiliations:** 1 Department of Hepatobiliary Surgery, Provincial Hospital affiliated to Shandong University, Jinan 250021, China; 2 Department of Oncology, Provincial Hospital affiliated to Shandong University, Jinan 250021, China

**Keywords:** BDH2, lncRNA, miR-141, metastasis, pancreatic cancer, TP73-AS1

## Abstract

LncRNA TP73 antisense RNA 1T (TP73-AS1) plays an important role in human malignancies. However, the levels of TP73-AS1 and its functional mechanisms in pancreatic cancer metastasis remain unknown, and the clinical significance of TP73-AS1 in human pancreatic cancer is also unclear. In the present study, the levels of TP73-AS1 and its candidate target miR-141 in pancreatic cancer and adjacent normal tissue were detected using qRT-PCR. The association between TP73-AS1 levels and the clinicopathologic characteristics of pancreatic cancer patients were analyzed. The relationship between TP73-AS1 and miR-141, and miR-141 and its candidate target 3-hydroxybutyrate dehydrogenase type 2 (BDH2) was confirmed using dual-luciferase reporter assays. TP73-AS1 and/or miR-141 were knocked down using siRNA or an inhibitor in pancreatic cancer cells and cell migration and invasion then examined. The results showed that TP73-AS1 was up-regulated in pancreatic cancer tissue and cell lines. High levels of TP73-AS1 were correlated with poor clinicopathological characteristics and shorter overall survival. MiR-141 was a direct target for TP73-AS1, while BDH2 was a direct target for miR-141. The knockdown of TP73-AS1 significantly inhibited the migration and invasion of pancreatic cancer cells, while the miR-141 inhibitor significantly restored the migration and invasion. Therefore, TP73-AS1 positively regulated BDH2 expression by sponging miR-141. These findings suggest that TP73-AS1 serves as an oncogene and promotes the metastasis of pancreatic cancer. Moreover, TP73-AS1 could serve as a predictor and a potential drug biotarget for pancreatic cancer.

## Introduction

Pancreatic cancer is one of the most common malignant tumors worldwide [1,2]. At present, surgery is the primary form of treatment for patients with early pancreatic cancer, but those in the advanced stages of the disease have typically lost the opportunity to undergo surgery [3]. The prognosis for pancreatic cancer remains poor, with an overall 5-year survival rate of less than 5% [1,2]. Although multiple genetic and epigenetic changes have been identified in pancreatic cancer, the exact pathogenesis of pancreatic cancer remains unclear [4]. Therefore, it is of great practical significance and theoretical value to study the occurrence and development of pancreatic cancer in order to discover novel and effective diagnosis and therapeutic biotargets.

Non-coding RNA (ncRNA) is ubiquitous in organisms and plays an important role in many cellular activities, though it cannot encode proteins and is not directly involved in protein synthesis. A number of forms of ncRNA are found in a wide variety of organisms, including micro RNA (miRNA), guide RNA, Piwi-interacting RNA, and long ncRNA (lncRNA). LncRNA, which has a length greater than 200 nucleotides [5], is widely distributed within the genes of humans. Although lncRNA does not encode proteins, it is involved in the complex and very important gene expression regulatory network [6]. Recent studies have shown that lncRNA plays an important role in normal tissue development, cell differentiation, and cancer metastasis.

Increasing evidence have shown that the aberrant expression of lncRNA is closely related to various human cancers including pancreatic cancer. LncRNA P73 antisense RNA 1T (TP73-AS1) is located on chromosome 1p36.32 of the human genome. It has been reported that lncRNA TP73-AS1 is overexpressed in non-small lung cancer [7], esophageal squamous cell carcinoma [8], gastric cancer [9–11], glioblastoma [12,13], glioma tissue [14], hepatocellular carcinoma [15], breast cancer [16,17], colorectal cancer [18], and ovarian cancer [19], but is down-regulated in bladder cancer tissue [20]. It has also been found that the knockdown of lncRNA TP73-AS1 attenuates the proliferation of esophageal cancer cells and enhances their chemosensitivity by inhibiting the expression of 3-hydroxybutyrate dehydrogenase type 2 (BDH2) [8]. The knockdown of lncRNA TP73-AS1 also reduces the expression of RFX1 and induces the apoptosis of glioblastoma multiforme cells [12], while TP73-AS1 promotes brain glioma growth and invasion by down-regulating HMGB1 via the sponging of miR-142 [14]. Previous research also indicates that TP73-AS1 competes with HMGB1 for miR-200a binding in hepatocellular carcinoma cells [15] and promotes breast cancer cell proliferation by acting as competing endogenous RNA (ceRNA) in sponging miR-200a [16,17]. Despite this volume of information, the role of TP73-AS1 in pancreatic cancer remains unclear.

In the present study, we monitored the expression of TP73-AS1 in pancreatic cancer tissue, and its association with the progression and poor overall survival rate of pancreatic cancer. We predicted that miR-41 was a candidate target for TP73-AS1 using bioinformatic analysis. MiR-141 is associated with the poor survival prospects of pancreatic cancer patients and its down-regulation is known to inhibit pancreatic cancer metastasis [21,22]. Thus, we also studied the interaction between TP73-AS1 and miR-141 in pancreatic cancer cell migration and invasion. Our results suggest that TP73-AS1 could serve as a predictor and a potential drug biotarget for pancreatic cancer.

## Materials and methods

### Pancreatic cancer specimens

Seventy-seven pairs of primary pancreatic cancer and adjacent normal tissue were obtained from patients who underwent surgery at Shandong Provincial Hospital affiliated to Shandong University between 2012 and 2017. Their pathological characteristics were recorded. The present study was approved by the Ethics Committee of Shandong University. The research was carried out in accordance with the World Medical Association Declaration of Helsinki, and all subjects provided written informed consent.

### Cell culture and treatment

The pancreatic duct epithelial cell line HPDE6-C7 and human pancreatic cancer cell lines SW1990, CAPAN-1, JF305, PANC-1, and BxPC-3 were purchased from ATCC, U.S.A. and were cultured in DMEM (Gibco, U.S.A.) supplemented with 10% fetal bovine serum at 37°C. For TP73-AS1 knockdown, TP73-AS1 siRNA (5′-CCTGCTGCCTCTCCAAGAGACTGCTATTA-3′) [11] and a negative control were transfected into cells using Lipofectamine 2000 (Invitrogen, U.S.A.) according to the manufacturer’s manual. MiR-141 mimics, inhibitors, and their negative controls were produced by RiboBio (Guangzhou, China).

### qRT-PCR

Total RNA was extracted using TRIzol reagent (Invitrogen, U.S.A.). cDNA was reversed using a PrimeScript RT reagent kit (Invitrogen). Polymerase chain reaction (PCR) analysis was performed using an ABI 7200 real-time PCR system (Applied Biosystems, U.S.A.) with an SYBR Green PCR Kit (Takara, Japan). The relative expression of detected genes was calculated using 2^−ΔΔ*C*^_t_. Three independent repetitions were carried out. The sequences of primers used were TP73-AS1: 5′-CCGGTTTTCCAGTTCTTGCAC-3′ (forward) and 5′-GCCTCACAGGGAAACTTCATGC-3′ (reverse), miR-141 5′-ACACTCCAGCTGGGCATCTTCCAG-3′ (forward) and 5′-AATTCAGTTGAGTCCAAC-3′ (reverse), and BDH2 5′-ACTGCCGAGCTCGTCGATCAAGAACTAAAG-3′ (forward) and 5′-ACGCTAAGGCTCTCATCTAGAAATATGTTC-3′ (reverse).

### Cell migration and invasion

Transwell chambers (Corning, U.S.A.) were used for both migration and invasion assessment. In the invasion analysis, a Transwell chamber was precoated with Matrigel (BD, U.S.A.). Cells were seeded in the upper chamber, and medium with 10% fetal bovine serum was added to the lower chamber. After 24 h, the cells that had not migrated or invaded were removed with a cotton swab. The cells on the lower surface were fixed with 4% paraformaldehyde, stained with 0.1% crystal violet, and counted.

### Luciferase reporter assays

The Dual-luciferase Reporter Assay System (Promega, U.S.A.) was employed according to the manufacturer’s manual. Wide-type or mutant TP73-AS1 with miR-141-binding sites was established, integrated into a pmir-GLO dual-luciferase vector, and cotransfected with miR-141 into cells using Lipofectamine 2000. After 48 h, luciferase activity was measured. Pmir-GLO-BDH2 wide-types or pmir-GLO-BDH2 mutants were created in a similar manner and cotransfected with miR-141 mimics or a negative control into cells. After 48 h, luciferase activity was measured.

### Western blot analysis

Proteins were lysed using RIPA (Beyotime, China), and the concentrations were measured using BCA protein assays (Beyotime, China). Proteins were separated using 10% SDS-PAGE and transferred to PVDF membranes (Millipore, U.S.A.). After incubation with primary antibodies against BDH2 and GAPDH (Abcam, U.S.A.) overnight at 4°C, the membranes were incubated with HRP-conjugated secondary antibodies at room temperature for 1 h. An ECL detection system (Millipore, U.S.A.) was used to analyze the specimens.

### Statistical analysis

The data were presented as the mean ± SD from at least three independent experiments. Statistical analysis was conducted with SPSS 17.0 (SPSS, U.S.A.). The association between TP73-AS1 and the clinicopathological characteristics of the patients with pancreatic cancer was analyzed using chi-squared tests. The overall survival curve was created using Kaplan–Meier survival analysis with log-rank testing. Student’s *t*-tests and one-way ANOVA with Turkey’s post-hoc test were conducted. A *P* value of less than 0.05 was considered statistically significant.

## Results

### TP73-AS1 is higher in pancreatic cancer tissue and cell lines

**Figure 1:**
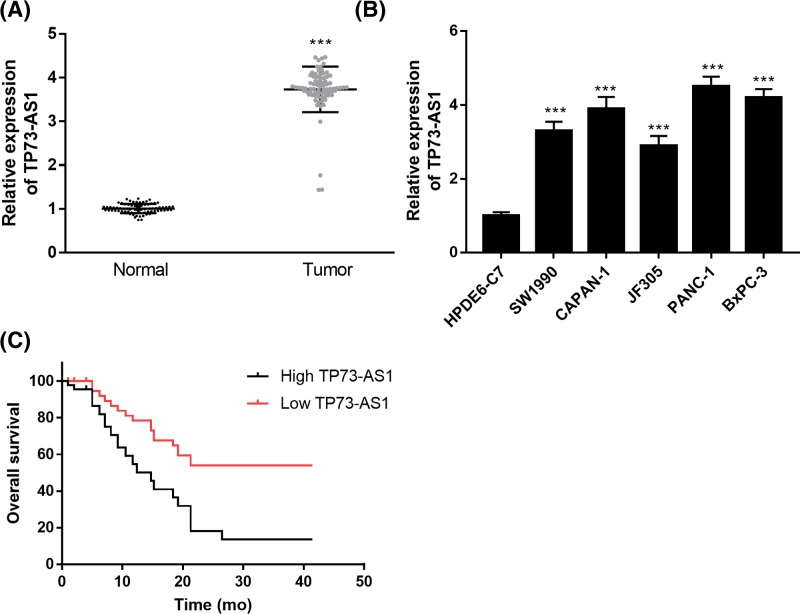
TP73-AS1 expression is up-regulated in pancreatic cancer tissues and cell lines (**A**) Expression of TP73-AS1 in pairs of pancreatic cancer and adjacent normal tissue (*n*=77). (**B**) Expression of TP73-AS1 in human pancreatic cancer cell lines and normal pancreatic cell line HPDE6-C7. (**C**) Overall survival of patients with pancreatic cancer. Kaplan–Meier analysis was performed (*P*<0.001); ***P<0.001.

The lncRNA TP73-AS1 was dysregulated in pancreatic cancer tissue and cell lines (Figure 1). The levels of TP73-AS1 in pancreatic cancer tissue and cells (SW1990, CAPAN-1, JF305, PANC-1, and BxPC-3) were significantly higher than in non-tumor normal tissue or normal pancreatic HPDE6-C7 cells (Figure 1A,B).

We also assessed the association between TP73-AS1 and the pathological characteristics of the pancreatic cancer patients (Table 1). The results showed that the overexpression of TP73-AS1 was significantly correlated with the TNM stage and lymph node metastasis, while no significant correlation was found between TP73-AS1 level and age or gender (Table 1). Based on Kaplan–Meier survival analysis, high expression levels of TP73-AS1 were found to be significantly associated with shorter overall survival in pancreatic cancer patients (*P*<0.001; Figure 1C).

**Table 1:** Relationship between TP73-AS1 and clinicopathological characteristics of pancreatic cancer patients

Features	TP73-AS1		*X*^2^	*P*
	High (*n*=45)	Low (*n*=32)		
Age (years)				
≥60	26	20	0.173	0.677
<60	19	12		
Gender				
Male	23	16	0.009	0.923
Female	22	16		
TNM stage				
I-II	15	19	5.143	0.023
III-IV	30	13		
Lymph node metastasis				
Negative	14	26	18.83	<0.001
Positive	31	6		

### Knockdown of TP73-AS1 inhibits the migration and invasion of pancreatic cancer cells

**Figure 2:**
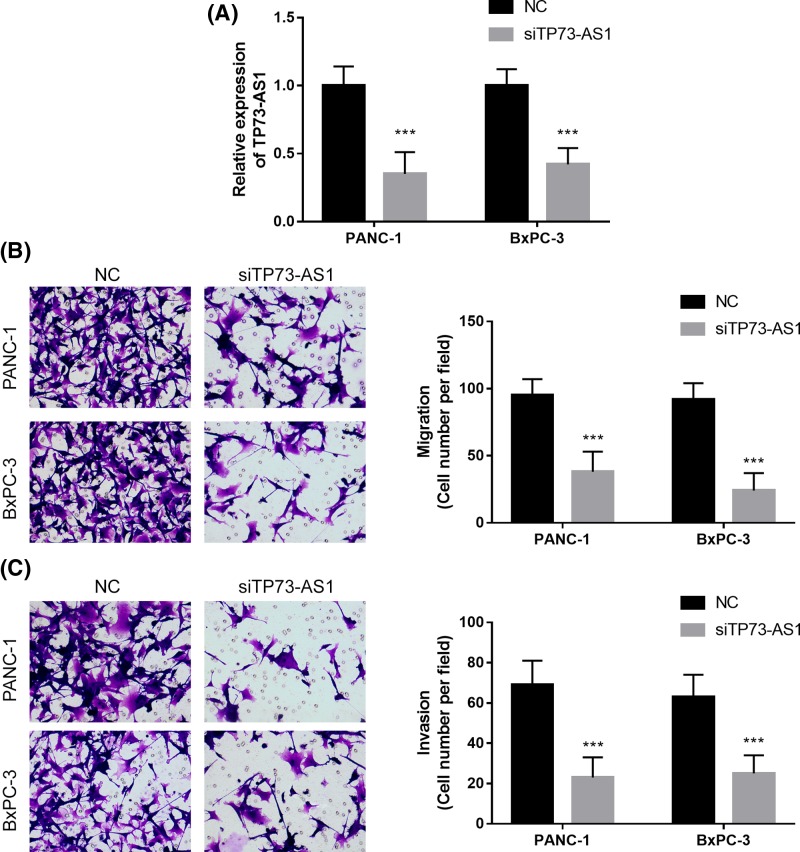
Knockdown of TP73-AS1 inhibits the migration and invasion of pancreatic cancer cells (**A**) Knockdown of TP73-AS1 by TP73-AS1 siRNA. (**B**) Migration assays were performed on transfected cells. (**C**) Invasion assays were performed on transfected cells; ***P<0.001.

To study the role of TP73-AS1 in pancreatic cancer metastasis, TP73-AS1 was silenced in both PANC-1 and BxPC-3 cell lines using TP73-AS1 siRNA (Figure 2A). The knockdown of TP73-AS1 inhibited migration and invasion in both the PANC-1 and BxPC-3 cell lines (Figure 2B,C). Thus, the knockdown of TP73-AS1 suppressed the metastasis of pancreatic cancer cells.

### TP73-AS1 knockdown inhibits cell metastasis through the direct interaction with miR-141

**Figure 3:**
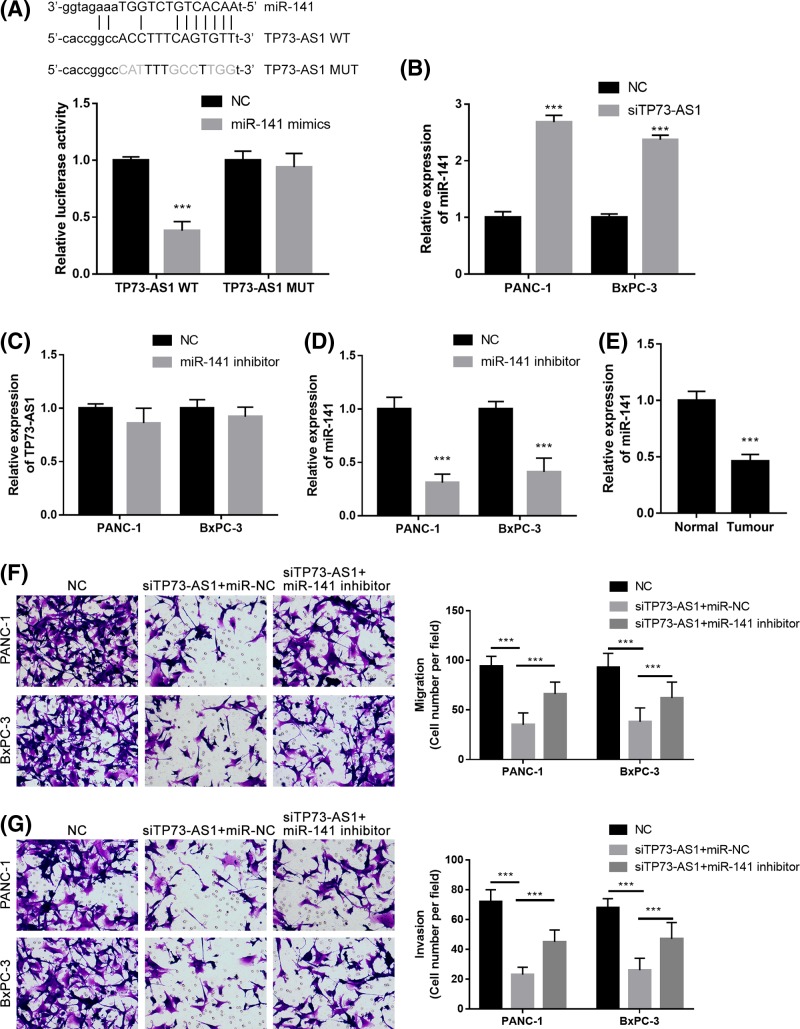
TP73-AS1 regulates the metastasis of pancreatic cancer cells through miR-141 (**A**) Prediction of binding sites between TP73-AS1 and miR-141; Luciferase reporter assays demonstrated that miR-141 mimics significantly decreased the luciferase activity of TP73-AS1 WT HEK293T cells but did not affect that of TP73-AS1 MUT. (**B**) Knockdown of TP73-AS1 in pancreatic cancer cells significantly increased the miR-141 expression. (**C**) TP73-AS1 was not significantly changed by the transfection of the miR-141 inhibitor in pancreatic cancer cells. (**D**) MiR-141 expression was down-regulated by the transfection of the miR-141 inhibitor in pancreatic cancer cells. (**E**) MiR-141 was significantly lower in pancreatic cancer tissue than in the adjacent normal tissue. (**F** and **G**) Migration (F) and invasion (G) assays for pancreatic cancer cells transfected with siTP73-AS1 in the presence of the miR-141 inhibitor or negative control; ***P<0.001.

Using the Starbase V2.0 database, miR-141 was identified as a potential ceRNA target for TP73-AS1. Dual-luciferase reporter assays showed that miR-141 mimics significantly reduced the luciferase activity of the TP73-AS1-wt luciferase reporter vector, while they did not affect the luciferase activity of the TP73-AS1-MUT luciferase reporter vector (Figure 3A). The knockdown of TP73-AS1 significantly increased miR-141 expression in both the PANC-1 and BxPC-3 cell lines (Figure 3B). The miR-141 inhibitor did not obviously change TP73-AS1 expression levels in either the PANC-1 or BxPC-3 cell lines (Figure 3C), while it significantly down-regulated miR-141 expression (Figure 3D). The expression levels of miR-141 were also significantly down-regulated in the pancreatic cancer tissue compared with the adjacent normal tissue (Figure 3E). Further experiments showed that the miR-141 inhibitor significantly reversed TP73-AS1-inhibited cell migration (Figure 3F) and cell invasion (Figure 3G) in both the PANC-1 and BxPC-3 cell lines. These results indicate that the up-regulation of TP73-AS1 is associated with the down-regulation of miR-141 in pancreatic cancer. The knockdown of TP73-AS1 thus suppresses cell metastasis by modulating miR-141.

### TP73-AS1 positively regulates BDH2 by regulating miR-141

**Figure 4:**
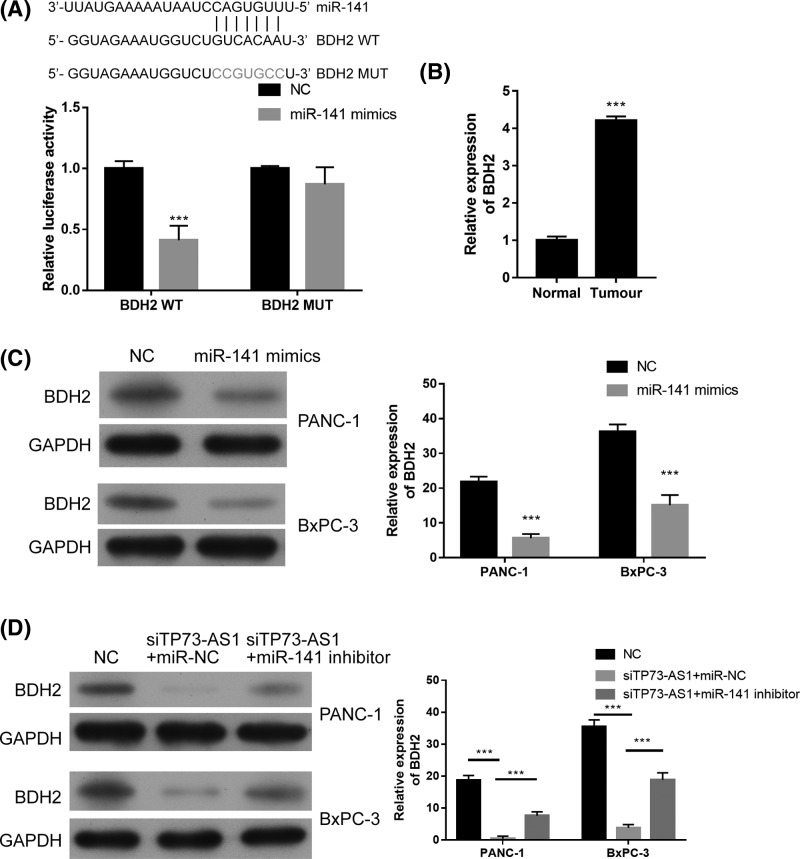
TP73-AS1 partially positively regulates BDH2 expression by inhibiting miR-141 in pancreatic cancer cells (**A**) Prediction of binding sites between miR-141 and BDH2; Luciferase reporter assays demonstrated that miR-141 mimics significantly decreased the luciferase activity of BDH2 WT HEK293T cells but did not affect that of BDH2 MUT. (**B**) BDH2 was significantly up-regulated in pancreatic cancer tissue compared with adjacent normal tissue. (**C**) BDH2 expression in pancreatic cancer cells transfected with miR-141 mimics. (**D**) BDH2 expression in pancreatic cancer cells transfected with siTP73-AS1 in the presence of the miR-141 inhibitor or negative control; ***P<0.001.

Using TargetScan, we screened BDH2 as a candidate target gene for miR-141 (Figure 4A). Luciferase reporter assays found that the overexpression of miR-141 significantly reduced the luciferase activity of BDH2-wt but did not obviously change that of BDH2-MUT (Figure 4A). BDH2 was also overexpressed in pancreatic cancer tissue when compared with the adjacent normal tissue (Figure 4B). The overexpression of miR-141 significantly inhibited the expression of BDH2 in both PANC-1 and BxPC-3 cells (Figure 4C). The knockdown of TP73-AS1 significantly reduced the expression of BDH2 in both PANC-1 and BxPC-3 cell lines, while the knockdown of miR-141 partially restored BDH2 expression (Figure 4D). MiR-141 inhibitor up-regulated BDH2 expression (Supplementary Figure S1). These results suggest that TP73-AS1 positively regulates BDH2 by regulating miR-141.

## Discussion

In the present study, we monitored the levels of TP73-AS1 in 77 pairs of primary pancreatic cancer and adjacent normal tissue. TP73-AS1 levels were significantly up-regulated in pancreatic cancer tissue and positively associated with TNM stage and lymph node metastasis in pancreatic cancer patients. In addition, the high expression of TP73-AS1 was significantly associated with lower overall survival. Tumor metastasis was assessed in terms of migration and invasion behavior [23,24].

The role of TP73-AS1 in various human cancers, including esophageal cancer, glioblastoma, and hepatocellular carcinoma, has been investigated in previous research [8,12,14], and it has been found to be particularly associated with miRNAs. For example, TP73-AS1 promotes tumor progression in gastric cancer by regulating miR-194-5p [25], while the down-regulation of TP73-AS1 inhibits triple-negative breast cancer cell vasculogenic mimicry by targeting miR-490-3p [26], and TP73-AS1 interacts with miR-124 to modulate glioma growth [27]. In the present study, we focused on the role of TP73-AS1 in the metastasis of pancreatic cancer tissue because it has been positively associated with lymph node metastasis in pancreatic cancer patients. The results showed that the knockdown of TP73-AS1 suppressed cell migration and invasion in pancreatic cancer cell lines, suggesting that TP73-AS1 acts as an oncogene in pancreatic cancer. We also evaluated the association between TP73-AS1 and its predicted target miR-141. MiR-141 is a member of the miR-200 family. Suppression of the transcription of miR-141 has been found to contribute to the activation of epithelial differentiation in pancreatic, colorectal, and breast cancer cells [28]. In the highly lymphatic metastatic pancreatic cancer cell line BxPC-3-LN, miR-141 is expressed at lower levels than in parental BxPC-3 cells, thus it may regulate lymphatic metastasis in pancreatic cancer [29]. Moreover, it has been reported that miR-141 acts as a tumor suppressor in pancreatic cancer cells [21]. We found that miR-141 serves as a direct target of TP73-AS1. The knockdown of TP73-AS1 significantly increased miR-141 expression levels, but the miR-141 inhibitor did not notably affect TP73-AS1 expression. However, the miR-141 inhibitor significantly reversed TP73-AS1-inhibited cell migration and cell invasion, suggesting that TP73-AS1 serves as a ceRNA by sponging miR-141.

MiR-141 can regulate cancer progression and metastasis through its targets. For example, miR-141 inhibits cell invasion by directly targeting MAP4K4 in pancreatic cancer [21], while it enhances apoptosis in PANC-1 cells by targeting Yes-associated protein-1 [30]. Here, we found that BDH2 is a direct target of miR-141. BDH2 is a member of the short-chain dehydrogenase/reductase family, which was originally referred to as dehydrogenase/reductase 6 (DHRS6) [31]. It has been found that BDH2 interacts with ferritin through its iron-responsive elements to control post-transcriptional gene expression, thus further confirming the physiological role of BDH2 in iron transport [33]. It may also play a crucial role in promoting tumor development [32]. Recently, Zughaier et al. [34] reported that the expression of BDH2 in macrophages was significantly inhibited due to bacterial infection, suggesting that BDH2 has an effect on iron-limited innate immunity. In addition, Yang et al. [35] found that BDH2 expression can be used as a new independent prognostic marker of cytogenetic normal acute myeloid leukemia, which has an anti-apoptotic effect. We found that BDH2 expression was significantly inhibited by miR-141 mimics and significantly induced by the miR-141 inhibitor. The knockdown of TP73-AS1 significantly reduced the expression of BDH2, while the knockdown of miR-141 partially restored BDH2 expression levels. These results suggest that TP73-AS1 positively regulates BDH2 by regulating miR-141.

In conclusion, we identified TP73-AS1 as an oncogene that plays a vital role in the metastasis of pancreatic cancer and found that it positively regulates BDH2 expression via the regulation of miR-141 expression in pancreatic cancer cells. As a result, TP73-AS1 shows promising effect as a potential therapeutic target for pancreatic cancer.

## Supporting information

**Supplementary Figure:** 

## Abbreviations

BDH2: 3-hydroxybutyrate dehydrogenase type 2
ceRNA: competing endogenous RNA
LncRNA: long ncRNA
ncRNA: non-coding RNA
