# Supplementary figures and images for: LncRNA TP73-AS1 sponges miR-141-3p to promote the migration and invasion of pancreatic cancer cells through the up-regulation of BDH2
Source: Biosci Rep. 2019 Mar 15;39(3):BSR20181937. doi: 10.1042/BSR20181937 (PMC6418400; doi:10.1042/BSR20181937)

|  | Blank | miR-NC | miR-141<br>inhibitor | miR-NC<br>+miR-141<br>inhibitor |
|--|-------|--------|----------------------|---------------------------------|
|--|-------|--------|----------------------|---------------------------------|

BDH2

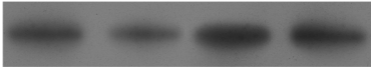

GAPDH

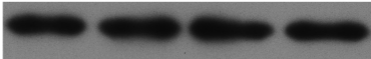

Supplement: Supplementary file 1 [file bsr-39-bsr20181937_Supp1.pdf]
